# Adult's Epstein–Barr virus‐associated hemophagocytic lymphohistiocytosis: A case report

**DOI:** 10.1002/ccr3.8117

**Published:** 2023-11-02

**Authors:** Yi Sun, Han Yao, Xiaofeng Yu, Yunzhao Chen, Chen Chai

**Affiliations:** ^1^ Department of General Surgery The People's Hospital of SND Suzhou China; ^2^ Department of Pathology The People's Hospital of SND Suzhou China

**Keywords:** case report, early diagnosis, Epstein–Barr virus, hemophagocytic lymphohistiocytosis, therapy

## Abstract

Adult's Epstein–Barr virus‐associated hemophagocytic lymphohistiocytosis is a rare and life‐threatening condition characterized by atypical initial symptoms and rapid disease progression. To facilitate early diagnosis and prompt treatment, it is imperative to implement early multidisciplinary intervention and prioritize pathogen detection, as these measures significantly contribute to enhancing patient prognosis.

## Graphical abstract

We herein report a misdiagnosed case of EBV‐HLH in an Asian adult and emphasize the importance of early multidisciplinary intervention and pathogen detection.

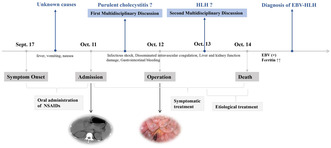

## 1 BACKGROUND

Hemophagocytic lymphohistiocytosis (HLH) is a life‐threatening syndrome characterized by excessive inflammation due to over‐activation of immune system. However, due to atypical early symptoms and a low incidence rate (0.9 cases per million persons), patients may not receive effective treatment in a timely manner, leading to a high mortality rate (about 30%).^1^, ^2^ Moreover, it has been observed that Asian patients are more susceptible to Epstein–Barr virus (EBV)‐HLH, with more than half of Asian HLH patients being infected with EBV.^3^, ^4^ We herein report a misdiagnosed case of EBV‐HLH in an Asian adult. Through analyzing the causes of misdiagnosis and reviewing relevant literature, we aim to highlight the current diagnostic challenges, therapeutic obstacles, and recent treatment advancements in EBV‐associated HLH.

## 2 CASE PRESENTATION

**FIGURE 1:**
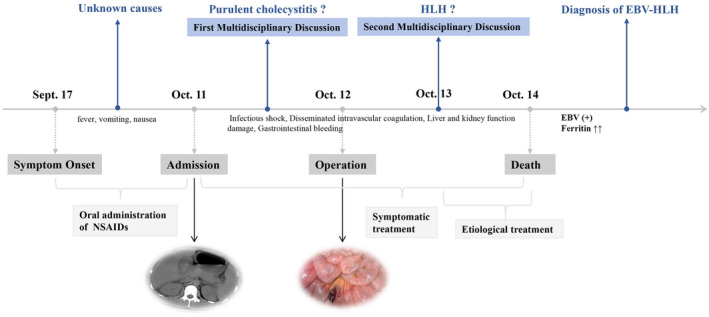
Timeline of interventions and outcomes.

**FIGURE 2:**
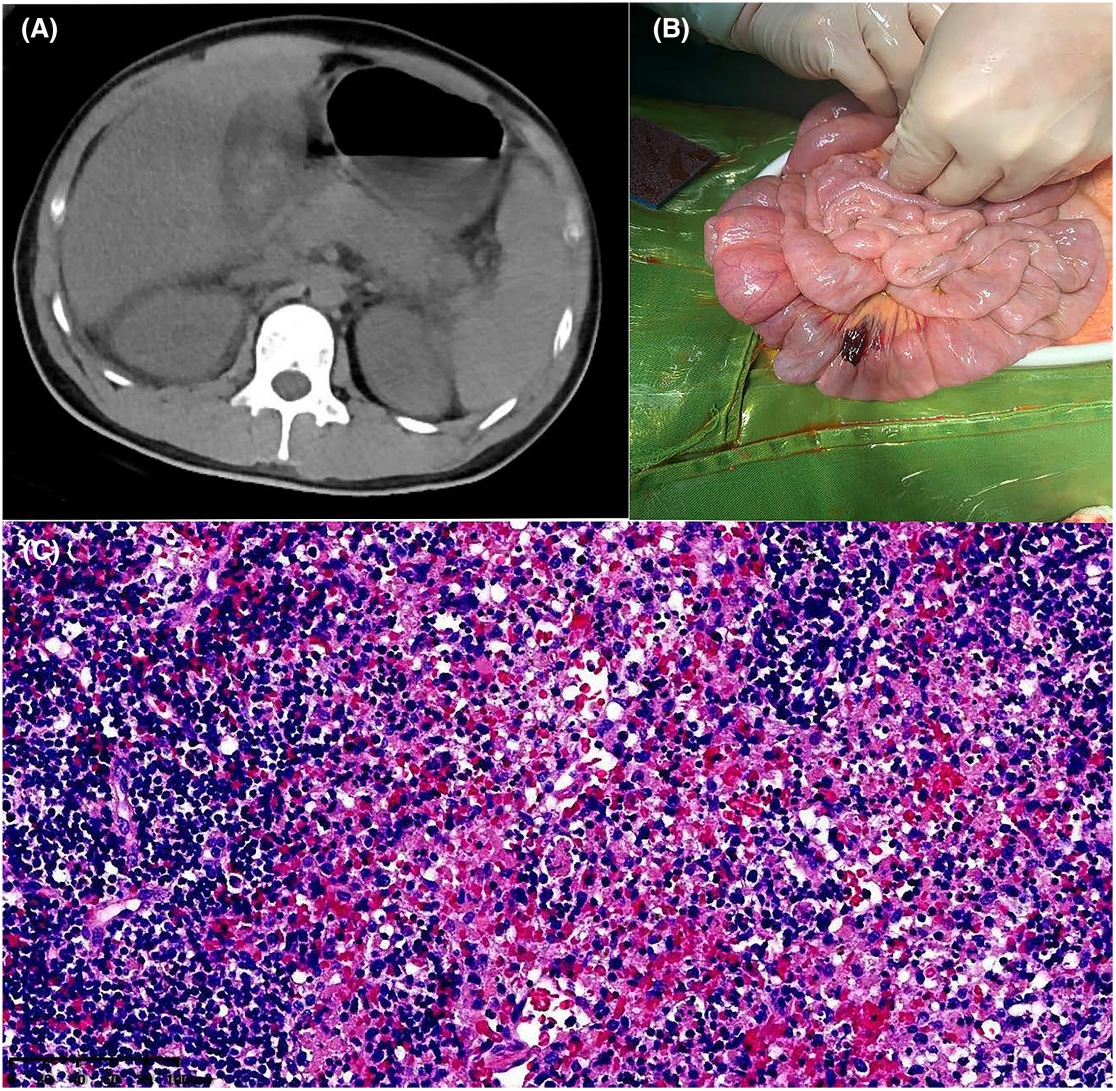
(A) Preoperative abdominal CT; (B) intraoperative findings of cholecystectomy; (C) postoperative pathology of gallbladder specimen.

On September 17th, a 21‐year‐old man presented with fever, vomiting, and nausea at his residence. He was not admitted to the Hematology Department of our hospital until October 11th, 2022 (Figure 1). Prior to admission, he had orally taken nonsteroidal anti‐inflammatory drugs (ibuprofen, 300 mg, twice daily) for over 2 weeks, but experienced minimal symptom relief. Moreover, this patient developed hematemesis and hematochezia upon admission. In addition to a high fever (>38.3°C), the patient exhibited apathy, xerocheilia, and upper‐middle abdominal pain with a heart rate of 155 beats per minute and blood pressure of 99/74 mmHg. Laboratory data (Table 1) indicated abnormal coagulation function, leukopenia, thrombocytopenia, and liver and kidney dysfunction. Thoracoabdominal computed tomography revealed scattered inflammation in the right lung, splenomegaly, and edematous and thickened gallbladder wall (Figure 2A). Based on the initial examination findings, the patient was diagnosed with infectious shock, disseminated intravascular coagulation, hepatic and renal dysfunction, and gastrointestinal bleeding. Therefore, the patient was transferred to the ICU for further treatment 8 h after admission. To identify potential pathogens, tests were conducted for immunocytes, cytokines, common pathogens, and blood bacterial culture. Furthermore, broad‐spectrum antimicrobial therapy with 1 g meropenem every 8 h was administered intravenously. Additionally, the treatment plan included the use of small‐molecule heparin (4100 IU, once daily), methylprednisolone (40 mg, once daily), dobutamine (6 μg/kg/min), norepinephrine (0.2 μg/kg/min), and albumin (25 g, qd), in accordance with other conventional therapies, including the application of organ‐protective medicines, alkalinizing, diuresis, and blood transfusion etc.

**TABLE 1:** Laboratory indicators on admission and the changes after treatment.

Measure	Reference range	Hospital Day 1	Hospital Day 2	Hospital Day 3	Hospital Day 4
APTT (s)	28–45	160	104.8	97.6	95.8
PT (s)	11–14.5	42.5	27.7	24.4	22.7
TT (s)	14–21	189	54.9	50.3	50.1
Fib (g/L)	2–4	<0.6	1.34	1.03	0.8
D‐D (μg/L)	0–0.5	>20	>20	>20	>20
WBC (×10^9^/L)	4–10	2.63	4.36	4.97	1.63
ANC (×10^9^/L)	2–7.5	1.87	3.76	4.47	1.3
ALC (×10^9^/L)	0.8–4	0.67	0.41	0.18	0.17
RBC (×10^9^/L)	3.5–5	4	3.19	2.55	1.72
Hb (g/L)	120–140	123	98	78	53
PLT (×10^9^/L)	125–350	31	25	24	18
PCT (ng/mL)	0–0.046	9.92	12.98	16.39	13.56
CRP (mg/L)	0–10	18.4	22.3	30.6	22.8
ALP (U/L)	35–104	150.7	123.2	110.7	136.5
AST (U/L)	5–40	1814	1105	1322.6	1402.6
ALT (U/L)	5–40	410	229.8	208.3	193.5
GGT (U/L)	5–60	168.7	249.5	160.5	111.2
Scr (μmol/L)	59–104	153.8	301.5	386.9	357
Albumin (g/L)	35–52	43.5	40.6	20.9	35.6
TB (μmol/L)	3.4–21	129.4	117.98	101.92	104.86
DB (μmol/L)	0–7	89.4	109.65	97.92	93.55
Hemodiastase (U/L)	35–135	76	124	133.9	106.9
Immunocytes^a^	—	Negative			
Cytokines^b^	—	Abnormal elevation			
Pathogen detection	—	Not available	Negative^c^		Positive^d^
Bacterial culture^e^	—	Not available			
Serum ferritin (ng/mL)	15–200	Not available			>2000.00

^**a**^ Antinuclear antibody (ANA), anti–smooth muscle antibody (SMA‐IgG), anti–mitochondrial M2 antibody (M2), anti–hepatorenal microsomal‐1 antibody (LKM‐1).

^b^ IL‐1β, IL‐12p, IL‐2/4/5/6/8/10/17, TNF‐α/β, α‐interferon.

^c^ Mycoplasma pneumoniae, *Legionella pneumophila*, *Rickettsia*, *Chlamydia pneumoniae*, adenovirus, respiratory syncytial virus, influenza A/B virus, parainfluenza virus, bocavirus, rhinovirus, metapneumonia virus (A & B).

^d^ Epstein–Barr virus, cytomegalovirus.

^e^ Bacterial culture were confirmed negative (October 17, 2022).

On the second day of admission, the patient exhibited slight improvement of symptoms, while blood test results showed signs of multi‐organ failure. We assessed that the risk of death for the patient was still high, and gangrenous cholecystitis was assumed to be the primary cause. After a multidisciplinary discussion, we decided to conduct an exploratory laparotomy and cholecystectomy. No clear evidence of intestinal necrosis was observed during the operation, but slight bleeding was detected in a spot of mesentery (Figure 2B). The edematous gallbladder exhibited two black spots, and black bile was extracted from the common bile duct. The pathological assessment indicated mild chronic inflammation, numerous necroses, phagocyte and sinus cell proliferation, and sporadic abnormal nuclear cells (Figure 2C). Postoperative blood test results showed slight improvement in the patient's blood coagulation and liver function. However, the blood lactate index increased slightly to 8 mmol/L, and the patient's blood pressure remained low at 92/46 mmHg, supported by vasopressors (norepinephrine at 0.1 μg/kg/min plus dobutamine at 8 μg/kg/min).

On the third day of hospitalization, a second multidisciplinary discussion was held, and rheumatologists were invited to participate. Further examination of respiratory pathogens, cytomegalovirus (CMV), EBV, and ferritin were recommended. Final results showed that the patient had a plasma EBV viral load of 3.66E+07 copies/mL and a CMV viral load below 1000 copies/mL. Ferritin levels were significantly elevated (>2000 ng/mL). Based on the patient's symptoms and examination results, we concluded that the patient was diagnosed with EBV‐induced HLH. Therefore, the treatment protocol was adjusted by employing continuous venous hemodialysis and administering high‐dose methylprednisolone. Given the patient's clear bleeding tendency, bone marrow puncture was deemed inadvisable. Sadly, the patient died due to shock after spending 4 days in the hospital without receiving the planned chemotherapy or hematopoietic stem cell transplantation (HCT).

## 3 DISCUSSION

Based on the presenting symptoms and pertinent examination findings, our patient fulfilled the HLH‐2004 diagnostic criteria, currently the most widely acknowledged diagnostic approach for HLH. Nonetheless, the delayed diagnosis somewhat reflects current dilemma in clinical practice: prompt initiation of multidisciplinary cooperation may not occur when HLH is encountered, and the diagnostic efficiency of HLH‐2004 criteria in adult's secondary HLH might be controversial. The patient's atypical symptoms did not provide strong indications of HLH, and this partly accounts for the delayed involvement of rheumatologists, as we initially believed the patient's symptoms were due to a biliary infection. Furthermore, parameters like ferritin, sIL‐2 r (sCD25), and NK‐cell activity, specified in HLH‐2004 diagnostic criteria, may not be routinely tested or available in standard laboratories. And it is noteworthy that HLH‐2004 diagnostic criteria stem from a single clinical trial that primed on pediatric patients with primary HLH.^5^ Consequently, its applicability to adult's or secondary HLH remains debatable. Recently, Fardet et al.^6^ have proposed that HScore may facilitate swifter diagnosis by utilizing uncomplicated evaluation parameters that determine an individual's risk of contracting HLH.

Despite the progress made in research, diagnosing and treating EBV‐HLH remains challenging. The patient exhibited fever, tachycardia, dyspnea, hepatomegaly, splenomegaly, and pancytopenia in the early stages. These atypical symptoms can easily be mistaken for manifestations of sepsis, lymphoma, and other infectious diseases. Consequently, morphologic, immunologic, genetic, and clinical criteria must be promptly considered in these situations.^7^ Early implementation of additional examinations such as bone marrow biopsy, 18F‐FDG PET, pathological and immunohistochemical examination, and karyotyping can aid in differentiating the diagnosis.^8^ The current primary therapeutic approach is the HLH‐94 protocol. Due to an initial misdiagnosis, we failed to administer sufficient amounts of glucocorticoids, cyclosporine, or etoposide in a timely manner. As reported by Mahlaoui et al.,^9^ a complete response was achieved in 73% of patients who received a therapeutic protocol including steroids and antithymocyte globulin. However, the early administration of cyclosporine may not be beneficial due to adverse effects such as hypertension and renal injury.^5^ Ramos's study suggests that using etoposide‐containing regimens could enhance survival rates in cancer and infection, particularly in cases of EBV infection.^10^ Some researchers suggest that plasma exchange and hematopoietic stem cell transplantation may be beneficial for HLH patients.^11^, ^12^

Epstein–Barr virus typically remains a latent infection without symptoms in its host. When EBV infections are symptomatic, they often appear as infectious mononucleosis, with severe lymphoproliferation being a rare occurrence.^13^ By abnormally proliferating B/T/NK cells, EBV may activate an abnormal systemic inflammatory response.^14^ Depending on differing clinicopathological features, these immune disorders can be classified as EBV‐HLH, chronic active EBV infections (CAEBV), and EBV‐associated T/NK‐cell lymphomas.^15^ EBV‐HLH, the most common type of EBV‐associated nonfamilial HLH, is thought to regulate immune system by inducing apoptosis and activating cytotoxic T lymphocytes.^16^ Previous research has confirmed that Fas (CD95) and perforin are involved in immune regulation during EB virus infections.^17^, ^18^ Thus far, the precise pathogenesis of EBV‐HLH is still unknown. Some studies have found that T cells express EBV CD21 on the cell surface and early lytic antigen expression deletion of EBV.^16^, ^19^ This may lead to EBV infection into T cells or NK cells and virus proliferation. Additionally, the latent membrane protein 1 of the EB virus can influence the apoptotic signaling of NK cells through the NF‐κB signaling pathway and microRNA regulation.^20^, ^21^ Lay et al. discovered that EBV infection of CD4^+^ T cells can enhance the production of tumor necrosis factor α and activate macrophages^22^ and similar phenomenon might occur in the case of CD8^+^ T cells. The concentration of cytochrome c and Fas in the serum of EBV‐HLH patients may indicate apoptosis of EBV‐infected cells.^23^ Moreover, the role of EBV genotypes and ethnic genetic differences has been research spots in EBV‐HLH, but breakthroughs have yet to be made.^24^ The relationship between EBV infection and the severity of EBV‐HLH remains unclear, and further research is needed to elucidate the pathogenic process of EBV‐HLH.

While antipathogen therapy is generally suitable, there is inadequate evidence to endorse the administration of anti‐EBV therapy.^25^ A study conducted by Meng et al.^26^ indicated that the employment of rituximab could potentially reduce EBV load and subsequently eliminate EBV‐carrying B cells in EBV‐HLH patients. However, EBV‐HLH often becomes relapsed or refractory due to incomplete clearance of the virus by etoposide‐based regimens. Therefore, HCT is often considered as a limited but effective method for treating refractory or relapsing HLH. Moreover, several treatments have exhibited benefits for refractory patients, such as ruxolitinib (JAK1/2 inhibitor), anakinra (IL‐1 blockade), alemtuzumab (anti‐CD52 antibody), and emapalumab (anti‐IFN‐g monoclonal antibody).^27^, ^28^, ^29^ Additionally, nivolumab, a programmed cell death‐1 (PD‐1) inhibitor, has been proven to be a valuable therapeutic option for treating relapsed or refractory EBV‐HLH.^30^ Recently, the development of clinical research on HLH has led to the emergence of more targeted drugs.

## 4 CONCLUSION

Clinicians are increasingly adopting standardized approaches for its diagnosis and management as understanding of HLH deepens. However, the challenge of early diagnosis and unknown pathogenesis often results in a delay in effective and targeted treatment of EBV‐HLH. To enable timely and precise diagnosis, implementation and updating of evidence‐based therapeutic protocols are essential. Furthermore, improving clinical outcomes for patients with EBV‐HLH mandates more high‐quality research to expose the mechanism of EBV‐HLH, and it is essential to encourage multidisciplinary cooperation.

## AUTHOR CONTRIBUTIONS

**Yi Sun:** Conceptualization; writing – original draft; writing – review and editing. **Han Yao:** Investigation. **Xiaofeng Yu:** Investigation. **Yunzhao Chen:** Data curation. **Chen Chai:** Conceptualization; writing – review and editing.

## FUNDING INFORMATION

This study was supported by the Technology Development Project of Suzhou City (SYSD2020084) and Scientific innovation fund of People's Hospital of Suzhou New District (SGY2020D01).

## CONFLICT OF INTEREST STATEMENT

The authors have no competing interests to declare that are relevant to the content of this article.

## ETHICS STATEMENT

The study was conducted in accordance with the Declaration of Helsinki and approved by the Ethics Committee of the People's Hospital of Suzhou New District (protocol code: 2022‐068; date of approval: 2022/11/17).

## CONSENT

Written informed consent was obtained from the patient to publish this report in accordance with the journal's patient consent policy.

## Data Availability

The datasets used and/or analyzed during the current study are available from the corresponding author on reasonable request.
